# Relationship between renal stone formation, mitral annular calcification and bone resorption markers

**DOI:** 10.4103/0256-4947.65264

**Published:** 2010

**Authors:** Ahmet Celik, Vedat Davutoglu, Kemal Sarica, Sakip Erturhan, Orhan Ozer, Ibrahim Sari, Mustafa Yilmaz, Yasemin Baltaci, Murat Akcay, Behcet Al, Murat Yuce, Necat Yilmaz

**Affiliations:** a From the Department of Biochemistry, Gaziantep University, School of Medicine, Gaziantep, Turkey; b From the Department of Cardiology, Gaziantep University, School of Medicine, Gaziantep, Turkey; c From the Department of Urology, Gaziantep University, School of Medicine, Gaziantep, Turkey; d From the Department of Nuclear Medicine, Gaziantep University, School of Medicine, Gaziantep, Turkey; e From the Department of Physiology, Gaziantep University, School of Medicine, Gaziantep, Turkey; f From the Department of Cardiology, Ataturk Research and Investigation Hospital, Ankara, Turkey; g From the Department of Emergency Medicine, Gaziantep University, School of Medicine, Gaziantep, Turkey

## Abstract

**BACKGROUND AND OBJECTIVES:**

Mitral annular calcification (MAC) is associated with osteoporosis and there is evidence of reduced bone mineral density (BMD) in patients with renal stone formation (RSF). Therefore, we designed this study to test if RSF was associated with MAC and if this association could be linked to bone resorption.

**METHODS:**

Fifty-nine patients (mean age, 41.5 years) with RSF and 40 healthy subjects (mean age, 44.2 years) underwent screening for MAC and BMD, and measuurements were taken of serum and urine electrolytes, parathyroid hormone, alkaline phosphatase and urine dypyridoline.

**RESULTS:**

MAC was diagnosed in 11 (18%) patients with RSF compared with 1 (2.5%) control (*P*=.01). Urine phosphorus, magnesium, sodium, potassium and chloride levels were lower (*P*<.001, *P*=.02, *P*<.001, *P*<.001 and *P*<.001, respectively), but serum alkaline phosphatase, calcium and potassium levels were higher (*P*=.008, *P*=.007 and *P*=.001, respectively) in patients with RSF versus those without RSF. None of these abnormalities were found in patients or subjects with MAC. Urine pyridoline levels were higher and T-scores were more negative (more osteopenic) in patients and subjects with MAC than in those without MAC (*P*=.01 and *P*=.004, respectively). In a multivariate analysis, only T-scores and urine dipyridoline level were predictive of MAC (*P*=.03 and *P*=.04, respectively).

**CONCLUSIONS:**

Screening for MAC and bone resorption markers in patients with RSF demonstrated a high incidence of MAC in these patients. The presence of MAC in patients with RSF was associated with bone resorption markers. This seemingly complex interrelationship between RSF, MAC and bone loss needs to be clarified in further studies.

Renal stone formation (RSF) is a disease that often affects young people. There is evidence of reduced bone mineral density (BMD) among patients who form renal stones.^1^,^2^ However, any etiopathogenetic relationship is still controversial. A recent review of studies on this topic revealed that renal stone-forming patients have decreased bone density that can be related, at least in part, to an abnormality in bone remodeling, although chronic low calcium intake may be a contributing factor.^3^ Mitral annular calcification (MAC) is a fibrous, degenerative calcification of the mitral valve annulus. A few studies have shown that MAC can be attributed to ectopic calcium deposits related to severe bone loss caused by osteoporosis.^1^,^2^

MAC was found in 26% of patients with chronic renal failure, which is related to abnormal calcium metabolism and an elevated calcium-phosphorus product.^4^ Furthermore, it has also been suggested that calcium released from bones is deposited in the mitral valve.^5^ In a recent study, we reported that MAC was associated with osteoporosis.^6^ Given that finding, we designed this study to test the primary hypothesis that patients with renal stones are likely to have increased bone resorption that may increase the risk of osteoporosis and also increases the risk of MAC. A secondary hypothesis was that the interrelationship between MAC and renal stone formation might be linked to bone resorption.

## METHODS

Consecutive patients diagnosed between January 2004 and March 2005 at our institution as having upper urinary stones composed of calcium oxalate were compared to age- and sex-matched control subjects for the presence of MAC and for differences in bone resorption markers. The control subjects were without a family history of renal stones or a medical history of nephrolithiasis or suspected renal colic, and renal stones were ruled out by ultrasonography. The controls were sampled randomly from the individuals who visited our institution for a health check-up. Smoking habit and presence of hypertension and diabetes were noted. Exclusion criteria were a history of renal insufficiency, a creatinine level more than 1.5 mg/dL, liver insufficiency, chronic urinary tract infection, diuretic usage, history of cancer, high suspicion of hyperparathyroidism, solitary kidney, medical record of renal tubular acidosis, medical record of any documented gastrointestinal disease, hyperuricemia, information regarding dietary intake indicating it differed substantially from the culture in the region, and hormone replacement therapy. This study was approved by the institutional review committee in our hospital, and informed consent was obtained from all subjects.

Biochemical variables included urine dipyridoline/urine creatinine (normal range: 3-7.4 nM/nM), urine sodium (range: 40-220 mEq/L), potassium (normal range: 25-125 mEq/L), urine chloride (normal range: 110-250 mEq/L), urine phosphorus (normal range: 40-130 mg/dL), and urine calcium (normal range <16 mg/dL), serum parathyroid hormone (normal range: 10-65 pg/mL), cholesterol (normal range: 50-200 mg/dL), high-density lipoprotein-cholesterol (normal range: 40-70 mg/dL), triglycerides (normal range: 60-200 mg/dL); low-density lipoprotein-cholesterol (normal range: 60-130 mg/dL), alkaline phosphatase (normal range: 53-128 U/L), serum sodium (normal range: 135-145 mEq/L), serum potassium (normal range: 3.5-5.1 mEq/L), serum magnesium (normal range: 1.7-2.4 mg/dL), serum calcium (normal range: 8.6-10.2 mg/dL), serum phosphorus (normal range: 2.5-4.5 mg/dL), and serum chloride (normal range: 98-107 mEq/L). Complete transthoracic echocardiography (TTE) studies were performed with a commercially available system (Acuson 128 XP/10C USA). The TTE criteria for MAC included an intense echo-protruding structure located at the junction of the atrioventricular groove and posterior mitral valve leaflet on the parasternal long-axis and apical 4-chamber view or on the parasternal short-axis view. In accordance with accepted grading,^7^ MAC was considered to be severe when the thickness of the intense echo-producing structure was ≥5 mm as measured with TTE in the 4-chamber or parasternal view. All studies were recorded on super-VHS tape and evaluated independently by 2 cardiologists with expertise in echocardiography. The observers who made the diagnosis of MAC were blinded to the measurement of other variables. The acceptable level of interobserver agreement in the results of the analysis of TTE was presented in our previous study (kappa=0.62).^6^

All patients underwent a bone mineral density (BMD) study with dual-energy X-ray absorptiometry (DXA). Measurements were performed in the anteroposterior view for the lumbar spine and proximal femur with a DXA scanner (Hologig QDR4500 Elite, Bedford, Mass). For their T-score values, the patients were grouped as having osteporosis, having osteopenia, or being normal according to the World Health Organization diagnostic criteria for osteoporosis defined in 1994.^8^ When the individual T-score value was ≤–2.5 at the spine or hip, osteoporosis was diagnosed; a T-score between –2.5 and –1 was classified as osteopenia; and a T-score ≥–1 was regarded as normal or healthy.

Data were analysed with SPSS software version 10.0 (SPSS, Inc., Chicago, Illinois). Numerical values were reported as the mean (standard deviation) or as a proportion of the sample size. Comparisons of characteristics of patients were made with either independent samples T-test for continuous variables or a Chi-square test or Fisher exact test for categorical data. Multivariate stepwise logistic regresssion analysis was used to estimate the relationship between RSF and MAC, bone resorption markers (urine pyridoline, T-scores/DXA) and to evaluate for potential confounders. In all analyses, a *P* value <.05 was considered to be statistically significant.

## RESULTS

The study included 59 patients with RSF with a mean (SD) age of 41.5 (14.9) years and 40 healthy subjects with a mean age of 44.2 (12.7) years. There were no differences between two groups in terms of age and sex (Table 1). MAC was diagnosed in 11 (18%) patients with RSF compared with 1 (2.5%) control (*P*=.01). Stone formation was significantly more common in patients with MAC compared with the controls (*P*=.01). The T-scores were not significantly different between patients with and without renal stones (*P*=.1). Urine pyridoline levels were higher (*P*=.01) and serum alkaline phosphatase levels lower (*P*=.01) in individuals with MAC versus those without MAC (Table 2). T-scores were more negative (more osteopenic) in those with MAC versus those without (*P*=.004). None of other parameters were significantly different between two groups (Table 2).

**Table 1:** Characteristics of patients and controls by presence of renal stones.

	Renal stone (n=59)	No renal stone (n=40)	*P*
Age (years) 4	41.5 (14.9)	39.2 (12.7)	NS
MAC (n and %)	11 (18)	1 (2.5)	.01
Urine dypiridoline (nM/L)	91.6 (63.8)	101.7 (46.3)	NS
Dypiridoline/creatinine	7.3 (1.3)	6.8 (1.1)	NS
Urine creatinine (mg/dL)	126.1 (71.1)	153.3 (62.7)	.05
Urine phosphorous (mg/dL)	36.8 (24.9)	60.1 (38.9)	<.001
Urine magnesium (mg/dL)	5.5 (3.5)	11.3 (19.1)	.02
Urine calcium (mg/dL)	10.9 (9.6)	14.3 (14.4)	NS
Urine sodium (mEq/L)	120.9 (57.1)	184.4 (69.5)	<.001
Urine potassium (mEq/L)	43.5 (22)	68.6 (24.5)	<.001
Urine chloride (mEq/L)	166.7 (85.5)	250.3 (95.1)	<.001
Serum PTH (pg/mL)	59 (51.3)	45.9 (19.3)	NS
Cholesterol (mg/dL)	168.1 (35.6)	155.04 (25.4)	.04
HDL-cholesterol (mg/dL)	33.2 (7.3)	37.1 (7.2)	.01
LDL-cholesterol (mg/dL)	100.2 (30.3)	86 (19.9)	.01
Triglyceride (mg/dL)	173.1 (102.5)	159.0 (102.9)	NS
Alkaline phosphatase (U/L)	194.7 (54.8)	166.2 (45.2)	.008
Serum Creatinine (mg/dL)	1.08 (05)	1.02 (03)	NS
Serum phosphorous (mg/dL)	3.1 (0.7)	3.3 (0.5)	NS
Serum magnesium (mg/dL)	2.1 (0.2)	2.1 (0.2)	NS
Serum calcium (mg/dL)	9.3 (0.5)	9.1 (0.4)	.07
Serum sodium (mg/dL)	137.4 (4.5)	136.4 (2.6)	NS
Serum potassium (mg/dL)	4.2 (0.7)	3.9 (0.2)	.01
Serum chloride (mg/dL)	101.7 (4.2)	103.3 (2.2)	.03
BMI	27.2 (4.6)	26.2 (5.8)	NS
T-score	-0.7 (1.3)	-0.4 (1.0)	NS

MAC: mitral annular calcification, PTH: parathyroid hormone, LDL: low-density lipoprotein, HDL: high-density lipoprotein.

**Table 2:** Characteristics of patients and controls by presence of mitral annular calcification.

	MAC (n=12)	No MAC (n=87)	*P*
Age (years)	40.08 (21.0)	38.3 (13.5)	NS
Urine dypiridoline (nM/L)	134.2 (77.3)	90.4 (52.4)	.01
Urine creatinine (mg/dL)	146.4 (79.5)	135.8 (67.7)	NS
Urine phosphorous (mg/dL)	41.8 (28.7)	46.8 (33.9)	NS
Urine magnesium (mg/dL)	6.8 (3.6)	8.0 (13.5)	NS
Urine calcium (mg/dL)	12.2 (8.4)	12.3 (12.2)	NS
Urine sodium (mEq/L)	138.6 (63.3)	147.6 (70.6)	NS
Urine potassium (mEq/L)	50.9 (26.6)	54.1 (26.1)	NS
Urine chloride (mEq/L)	213.6 (127.6)	198.7 (94.1)	NS
Serum PTH (pg/mL)	58.9 (27.9)	53.0 (43.4)	NS
Cholesterol (mg/dL)	168.0 (31.9)	162.1 (32.6)	NS
HDL-cholesterol (mg/dL)	32.9 (4.8)	35.0 (7.8)	NS
LDL-cholesterol (mg/dL)	96.1 (33.9)	94.2 (26.6)	NS
Triglyceride (mg/dL)	194.3 (136.7)	163.7 (97.1)	NS
Alkaline phosphatase (U/L)	219.2 (49.7)	178.4 (51.6)	.01
Serum phosphorous (mg/dL)	3.1 (0.6)	3.2 (0.6)	NS
Serum magnesium (mg/dL)	2.1 (0.1)	2.1 (0.2)	NS
Serum calcium (mg/dL)	9.2 (0.4)	9.2 (0.5)	NS
Serum sodium (mg/dL)	136.8 (2.2)	137.0 (4.1)	NS
Serum potassium (mg/dL)	4.1 (0.3)	4.1 (0.6)	NS
Serum chloride (mg/dL)	102.8 (3.3)	102.3 (3.6)	NS
T-score	-1.6 (1.0)	-0.5 (1.2)	.004

MAC: mitral annular calcification, PTH: parathyroid hormone, LDL: low-density lipoprotein, HDL: high-density lipoprotein.

In a stepwise logistic regression multivariate analysis that included age, T scores, urine electrolytes, serum electrolytes, urine creatinine, serum creatinine, serum alcalen phosphatase, serum parathormone, urine dipyridoline, only T-scores and urine dipyridoline level were predictive of MAC (*P*=.03 and *P*=.04, respectively).

## DISCUSSION

One goal of this study was to screen for MAC in renal stone forming patients. Another goal was to determine whether any relationship between MAC and RSF could be linked to bone resorption. To our knowledge, this study is the first to show a significant incidence of MAC in renal stone forming patients. Moreover, we found that the presence of MAC in renal stone forming patients was strongly and independently associated with bone loss assessed with bone resorption markers.

The evidence of reduced BMD among renal stone forming patients has been suggested in previous studies.^1^,^2^ In our recent study, we reported that MAC was associated with osteoporosis.^6^ This study confirms that MAC is associated with bone loss. Since most of MAC subjects were in the renal stone forming group, our hypothesis of a pathopysiologic linkage between renal stone, MAC and bone loss has been supported.^8^

Several studies have shown an association between MAC and abnormal calcium metabolism.^4^,^9^ MAC was found in 26% of patients with chronic renal failure and was described in a patient with chronic renal failure and an elevated calcium-phosphorus product.^4^ Chronic hypercalcemia is associated with accelerated deposition of calcium in the cardiac annulus and valvular cusps. Furthermore, it has been also suggested that calcium released from the bones is deposited in the mitral valve.^5^ In our study hypercalcemia was found in the renal stone forming group in comparison with the control group. This may be involved in the formation of MAC in the setting of bone loss, but the cause/effect relationship needs to be further evaluated. Although a defective tubular reabsorption of calcium with secondary hyperparathyroidism or a defective tubular reabsorption of phosphate with a low serum phosphate stimulating 1,25 vitamin-D synthesis could be a possible explanation for the low BMD observed in some patients with calcium nephrolithiasis,^3^ reduced bone mineral content has been reported in calcium renal stone formers without signs of secondary hyperparathyroidism, as in our study.

We found some electrolyte abnormalities related to RSF. The possible causes of urine electrolyte abnormality in RSF subjects may result from the following possible factors: abnormal urinary pH values and acid crystallization, which has been reported in renal stone formers, or increased urinary oxalate excretion and calcium oxalate supersaturation, which results in the formation of calcium oxalate kidney stones. Additionally, the functional abnormality of the terminal collecting ducts and impaired concentration mechanism may, in part, be related to the urine electrolyte abnormality in renal stone formers. Both univariate and multivariate analyses revealed that only T-scores and and urine pyridoline were predictive of MAC.

The pathogenetically unknown mechanistic association of dyslipidemia and stone formation has been demonstrated in some studies.^10^ Our study supports this association. We found statistically significant dyslipidemic profile in the renal stone forming group. Oxidized lipids, osteoprotegerin, and bisphosphonates appear to regulate mineralization in both bone and vasculature and this may account for the coexistance of osteoporosis and vascular calcification.^11^

There are several limitations in the present study. The sample was relatively small, but the major limitation was selection bias. We did not measure glomerular filtration rate. We can not extend our study results to a broader population of patients with renal stones. However, our results offer a rationale for further studies assessing the mechanistic interrelationship between MAC and RSF. Our findings raise the question of whether treatment strategies targeting bone loss may affect RSF and MAC. However, our study is cross-sectional and therefore we cannot comment on a causal relationship. This needs to be assessed in further studies.

In conclusion, screening for MAC and bone resorption markers in renal stone forming patients demonstrated a high incidence of MAC in patients with renal stone, and the presence of MAC in these patients was strongly associated with bone resorption markers. The complex interrelation between RSF, MAC and bone loss needs to be clarified in further studies.
